# Association of a Patient’s Type of Insurance With Preventive Service Delivery

**DOI:** 10.7759/cureus.44927

**Published:** 2023-09-08

**Authors:** Cuneyt Ozkardes, Jeffrey Harman PhD

**Affiliations:** 1 Research, Florida State University College of Medicine, Tallahassee, USA; 2 Behavioral Health, Florida State University College of Medicine, Tallahassee, USA

**Keywords:** screening, uspstf, chronic disease, patient insurance, prevention

## Abstract

Introduction

Chronic disease or death associated with chronic disease is often avoidable with proper interventions, including preventive services in healthcare settings. However, preventive service delivery rates by physicians are low. This study examined the association between preventive services and the type of patient insurance, as well as the association between the duration of an office visit and the type of patient insurance.

Methods

A retrospective observational cohort study was conducted using multivariate logistic regression. Pooled data on physician office-based visits from the 2011-2016 and 2018 National Ambulatory Medical Care Survey (NAMCS) were used in this analysis. The main measures observed were the odds of providing preventive services as they related to the type of patient insurance. Preventive measures assessed were those recommended by the United States Preventive Services Task Force with an A or B grade, applicable to most adults 18-64. Additionally, the mean office visit duration was analyzed based on the type of insurance.

Results

The odds of receiving cervical cancer screenings and tobacco smoking cessation screenings were 47% (p <0.01) and 31% (p =0.10) lower with Medicaid than private insurance, respectively. The odds of receiving blood pressure screenings and cervical cancer screenings were 43% (p =0.01) and 62% (p <0.01) lower in uninsured office visits compared to private insurance, respectively. Compared to private insurance, Medicaid and uninsured visits were 2.68 minutes and 1.97 minutes shorter in duration, respectively (p <0.05).

Conclusions

An association was found between the type of patient's insurance and the odds of providing preventive services. If the aim of healthcare is to improve the incidence of chronic disease or death associated with chronic disease, preventive services should be provided regardless of insurance type.

## Introduction

As of 2014, 60% of Americans had at least one chronic condition, while 42% had multiple chronic conditions [1]. Americans with chronic diseases spend more on healthcare services and can have reduced physical and social functioning [2]. Although highly prevalent, chronic disease or death associated with chronic disease is often avoidable with proper interventions, including preventive services in healthcare settings [3]. Prevention for chronic disease emphasizes early disease detection and targets healthy-appearing individuals with subclinical forms of the disease, often in the form of screenings or counseling [4]. Preventive services have been effective in reducing the incidence of a targeted disease or condition and helping people live longer, healthier lives [5].

Since 1984, one of the nation’s leading panels of experts in preventive care has been the U.S. Preventive Services Task Force (USPSTF or Task Force) [6]. The Task Force was put forth by the U.S. Congress as an independent body of experts in prevention and evidence-based medicine. The Task Force has been responsible for making recommendations about clinical preventive services such as screenings, counseling services, or preventive medications. The measures for preventive care issued by the Task Force can be delivered or referred to by primary care providers. Preventive care guidelines from the USPSTF promote health, longevity, and improved quality of life. Maciosek and colleagues estimated that greater use of proven clinical preventive services, such as those recommended by the USPSTF, could produce more than 2,000,000 years of life annually without an increase in net cost [7].

Most studies about the delivery of preventive services predate the current landscape of healthcare in the United States. In 2010, a significant change was made in healthcare with the introduction of the Affordable Care Act (ACA), which required healthcare plans through the marketplace to cover preventive services without charging patients a copayment or coinsurance [8]. Prior to the ACA, healthcare plans were not required to cover preventive services. This change modified the role of patients in the healthcare setting as it pertains to the delivery of services. Specifically, patients have the right to demand ACA-mandated services from their healthcare plans. Although the ACA mandates healthcare plans to cover preventive services, various insurance plans reimburse physicians at different rates for the same services. A study on the use of preventive services among Medicaid enrollees showed no significant differences in the use of preventive services compared to patients with private insurance [9]. However, this study used data from 2003 and 2008, which predated the ACA. As of 2016, Medicaid programs paid physicians at 72% of Medicare rates [10].

Although the importance and effectiveness of preventive services have been demonstrated, their use in clinical settings is questionably low. As of 2015, only 8% of adults 35 and older in the U.S. received all high-priority, appropriate clinical preventive services. Nearly 5% of adults 25 and older in the U.S. did not receive any services [11]. Previous studies have explored why clinical preventive service rates are low. Financial incentives, reimbursement structures, and lack of time have been shown to significantly influence physicians' supply of health care and preventive services [12-14]. Levine and colleagues reported that physicians did not prioritize preventive care services, although they knew preventive services could reduce the incidence and burden of chronic diseases [1]. They found the major reason physicians did not prioritize preventive care services was because financial incentives did not align with a focus on preventing chronic diseases. A recent study found the odds of providing certain preventive services in office visits varied based on how physicians are compensated, suggesting financial incentives play a role in the delivery of care. [15]

Lack of physician time has also been identified as a major contributor to shortfalls in the delivery of recommended healthcare services [16]. Yarnall and colleagues concluded it would take 7.4 hours per working day for physicians to fully satisfy USPSTF recommendations for the provision of preventive services [17]. A recent study reported variations in the duration of office visits based on how physicians were compensated. The duration of office visits with physicians on productivity-based compensation models (i.e., fee-for-service) was 4.6 minutes shorter, on average, compared to salaried physicians [15]. Additionally, variation in office visit duration could be related to lower reimbursement rates from Medicaid and uninsured patients, with physicians addressing this by shortening these visits. However, shorter office visits will likely reduce the likelihood that physicians will provide the full suite of preventive services to these patients.

Patients with private and public insurance report higher rates of routine care than uninsured patients, and a lack of health insurance is associated with a lower probability of receiving recommended services that are due during a clinic visit [18,19]. A 2011 study showed the percentage of physicians accepting new patients with Medicaid was lower than the percentage accepting new self-pay, Medicare, or privately insured patients [20]. Decker also reported that higher state Medicaid-to-Medicare fee ratios were correlated with greater acceptance of new Medicaid patients, which aligned with prior suggestions that physicians are more likely to accept Medicaid patients when Medicaid reimbursement rates increase [21]. She also found that the increase in Medicaid reimbursement rates led to physicians spending more time with those patients during office visits [22]. This study assessed the association between the type of patient insurance, the delivery of preventive care services, and the duration of an office visit. It was hypothesized that uninsured patients and those on Medicaid were less likely to receive preventive services compared to patients with private insurance and have shorter office visits due to differences in reimbursement rates.

## Materials and methods

Sample

This study used pooled data from the 2011-2016 and 2018 National Ambulatory Medical Care Survey (NAMCS), a nationally representative survey sample conducted by the National Center for Health Statistics annually of office visits to physicians throughout the United States. The study was conducted at Florida State University College of Medicine, Tallahassee, United States. At the time of this study, the 2017 survey had not been made available to the public. The office visits included in the NAMCS are those delivered as outpatient care in freestanding, office-based practices, including health maintenance organizations and non-federal government clinics. Physicians were asked to record information on a standardized form for office visits made over a randomly selected one-week period, including information about the major reason for the patient’s visit, whether he or she identified as the patient's primary care physician, the type of office visit, and the treatments or services provided. A detailed description of the NAMCS survey and sampling strategies can be found elsewhere [23].

Office visits were limited to primary care specialties or providers that self-identify as the patient’s primary care providers, those that listed the major reason for the visit as ‘preventive care’, and patients aged 18-64. Due to the small number of office visits where the patient’s insurance was Medicare or workers’ compensation, these visits were excluded from this study. The sample consisted of 16,350 office visits. Of the 16,350 office visits, 12,587 were private insurance, 3,094 were Medicaid, the Children's Health Insurance Program (CHIP), or other state-based programs, and 669 were self-pay, no charge, or charity (Table 1).

**Table 1:** Patient demographics *p <0.05 CI: confidence interval; OB/GYN: obstetrics/gynecology

	All visits n %	Private n%	Medicaid n%	Uninsured n%
Patient visits (n raw)	16,350	12,587 (77%)	3,094 (19%)	669 (4%)
Patient visits (n weighted)	316,379,883	234,522,770	72,154,372	9,702,740
Age (years)	38.8	40.8	30.4	39.3
Sex				
Male	3,253 (20%)	2,744 (22%)	280 (9%)	229 (34%)
Female	13,097 (80%)	9,843 (78%)	2,814 (91%)	440 (66%)
Race-Ethnicity				
Non-Hispanic White	11,417 (70%)	9,536 (76%)	1,487 (48%)	394 (59%)
Non-Hispanic Black	1,930 (12%)	1,142 (9%)	702 (23%)	86 (13%)
Hispanic	2,062 (13%)	1,172 (9%)	757 (24%)	133 (20%)
Non-Hispanic other	941 (6%)	737 (6%)	148 (5%)	56 (8%)
Physician speciality category				
General and family practice	4,359 (28%)	3,422 (27%)	669 (22%)	268 (40%)
Internal medicine	2,338 (15%)	2,010 (16%)	209 (7%)	119 (18%)
Pediatrics	347 (2%)	274 (2%)	56 (2%)	17 (3%)
OB/GYN	8,328 (53%)	6,122 (49%)	1,979 (64%)	227 (34%)
Other	294 (2%)	261 (2%)	21 (1%)	12 (2%)
Unknown	684 (4%)	498 (4%)	100 (3%)	26 (4%)
Visit duration (minutes)* [95% CI]	21.4 [20.9, 22.0]	22.0 [21.5, 22.6]	19.4 [18.4, 20.3]	20.1 [18.4, 21.7]
Difference (minutes)* (weighted) [95% CI]	--	1.0 [Reference]	-2.68 [-3.72, -1.64]	-1.97 [3.67, -0.27]

Procedures

For this study, the 102 preventive service recommendations published (as of June 29, 2021) by the United States Preventive Services Task Force (USPSTF or Task Force) were reviewed. The 102 recommendations were narrowed to eight preventive services applicable to the majority of adults aged 18-64. Only currently active, recommended preventive services were selected to be included in this study. Of the active recommendations, those with a Task Force grade of ‘A’ or ‘B’ were chosen. Task Force recommendations given an ‘A’ rating are defined as services with a high certainty that the net benefit of the service is substantial. Task Force recommendations given a ‘B’ rating are defined as services with high certainty that the net benefit is moderate or with moderate certainty that the net benefit is moderate to substantial. Finally, only recommendations categorized as ‘screening’ or ‘counseling’ interventions were used for this study. This resulted in a final list of eight preventative services. These eight recommendations are applicable and generalizable to the majority of adults aged 18-64 (Table 2).

**Table 2:** Selected Task Force descriptions Adapted from the U.S. Preventive Services Task Force [24] OBPM: office blood pressure measurement; USPSTF: U.S. Preventive Services Task Force; IPV: intimate partner violence; BMI: body mass index; FDA: U.S. Food and Drug Administration

Service	Grade	Intervention	USPSTF Recommendation/Population
High blood pressure	A	Screening	“Screening for hypertension in adults 18 years or older with office blood pressure measurement (OBPM). The USPSTF recommends obtaining blood pressure measurements outside of the clinical setting for diagnostic confirmation before starting treatment.”
Depression	B	Screening	“Screening for depression in the general adult population, including pregnant and postpartum women. Screening should be implemented with adequate systems in place to ensure accurate diagnosis, effective treatment, and appropriate follow-up.”
HIV infection	A	Screening	“Screening for HIV infection in adolescents and adults aged 15 to 65 years. Younger adolescents and older adults who are at increased risk of infection should also be screened.”
Unhealthy alcohol use	B	Screening	“Screening for unhealthy alcohol use in primary care settings in adults 18 years or older, including pregnant women, and providing persons engaged in risky or hazardous drinking with brief behavioral counseling interventions to reduce unhealthy alcohol use.”
Cervical cancer	A	Screening	“Screening for cervical cancer every three years with cervical cytology alone in women aged 21 to 29 years. For women aged 30 to 65 years, the USPSTF recommends screening every three years with cervical cytology alone, every five years with high-risk human papillomavirus (hrHPV) testing alone, or every five years with hrHPV testing in combination with cytology (cotesting).”
Intimate partner violence	B	Screening	“Screening for intimate partner violence (IPV) in women of reproductive age and provide or refer women who screen positive to ongoing support services.”
Weight-loss	B	Counseling	“Offer or refer adults with a body mass index (BMI) of 30 or higher (calculated as weight in kilograms divided by height in meters squared) to intensive, multicomponent behavioral interventions.”
Tobacco smoking cessation	A	Counseling	“Ask all adults about tobacco use, advise them to stop using tobacco, and provide behavioral interventions and US Food and Drug Administration (FDA)--approved pharmacotherapy for cessation to nonpregnant adults who use tobacco.”

Four of the eight services examined in this study were applicable to the majority of visits by adults aged 18-64. However, for the remaining four services, the visits were further limited to match the applicable population, such as females or body mass index values, as specified by the Task Force. Adjusting these sample populations was done to ensure the samples represented only the population to which the Task Force recommends the service be delivered. Cervical cancer screening, intimate partner violence, weight loss to prevent obesity-related morbidity and mortality counseling, and tobacco cessation counseling were the four services with adjusted samples, and their population sizes were n = 12,277; n = 4,521; n = 3,558; and n = 1,731, respectively. The cervical cancer screening was adjusted to only include visits by females aged 21-64 (excluding visits by all males and females 18-20 years old). The intimate partner violence screening was adjusted to only include visits by females of reproductive age, which was defined in this study as women 18-55 years old (excluding visits by all males and females aged 56-64). The weight loss to prevent obesity-related morbidity and mortality counseling was adjusted to only include visits by adults with a body mass index (BMI) of 30 or higher (excluding visits by all adults with a BMI below 30). The tobacco cessation counseling was adjusted to only include visits by current smokers. Table 1 displays the chosen services used in this study along with their definitions and designated populations.

Multivariate logistic regression was used to compare the odds of receiving each selected preventive service for visits by patients with private insurance, Medicaid, CHIP, or other state-based programs, self-pay, or no charge or charity. The logistic regression models controlled for patients' age, gender, and race/ethnicity. The NAMCS states that for all estimates other than estimates of proportion, at least 30 observations should be present in order to produce stable estimates. Certain services examined in this study contained fewer than 30 observations and, therefore, were not prevalent enough to make stable estimates and were excluded from the analysis.

Analysis

All analyses used the survey procedures of Stata SE, version 17.0, to allow for estimates to be nationally representative and for standard errors to correctly account for the complex sampling strategy of the NAMCS, with 95% confidence intervals (CIs) calculated by using these weights for all estimated odds ratios (ORs). This study was declared exempt by the Florida State University Institutional Review Board.

## Results

The purpose of this study was to examine whether patient insurance was associated with preventive services provided during preventive care office visits. The study looked at 16,350 office visits during the years 2011-2016, and 2018 by patients to primary care providers. Of the 16,350 office visits, 77% were covered by private insurance, 19% were covered by Medicaid, and 4% were uninsured. Of the office visits examined in this study, 28% were to general and family practice physicians, 15% to internal medicine specialists, 53% to obstetrics/gynecology (OB/GYN) specialists, 2% to pediatricians, 2% to other specialists, and 4% were unknown (Table 1).

The unweighted number of visits with each type of insurance is depicted in Table 3. Four of the eight preventive services observed applied to the entire sample (n= 16,350) (unhealthy alcohol use and intimate partner violence were not documented until 2014), whereas the other four services applied to only a subset of the sample. Cervical cancer screening, intimate partner violence screening, weight loss to prevent obesity-related morbidity and mortality counseling, and tobacco smoking cessation counseling were the four services with adjusted samples.

**Table 3:** Data of received services (unweighted) The National Ambulatory Medical Care Survey (NAMCS) states for all estimates, other than estimates of proportion, at least 30 observations should be present. 1.  These services were not provided until 2014, therefore, the sample sizes are significantly smaller than other services.

Service	All Visits (n= 16,350) n %	Private (n= 12,048) n %	Medicaid (n= 3,094) n %	Uninsured (n= 669) n %
High blood pressure	15,305 (94%)	11,845 (94%)	2,880 (93%)	580 (87%)
Depression	641 (4%)	539 (4%)	79 (3%)	23* (3%)
HIV infection	417 (3%)	284 (2%)	113 (4%)	20* (3%)
	All visits (n= 6,389) n %	Private (n= 4,906) n %	Medicaid (n= 1,288) n %	Uninsured (n= 195) n %
Unhealthy alcohol use^1^	116 (2%)	97 (2%)	15* (1%)	4* (2%)
	All visits (n= 12,277) n %	Private (n= 9,445) n %	Medicaid (n= 2,423) n %	Uninsured (n= 409) n %
Cervical cancer	3,019 (25%)	2,633 (28%)	307 (13%)	79 (19%)
	All visits (n= 4,521) n %	Private (n= 3,258) n %	Medicaid (n= 1,151) n %	Uninsured (n= 112) n %
Intimate partner violence^1^	66 (1.5%)	48 (1.5%)	16* (1.4%)	2* (1.8%)
	All visits (n= 3,558) n %	Private (n= 2,867) n %	Medicaid (n= 537) n %	Uninsured (n= 154) n %
Weight loss to prevent obesity-related morbidity and mortality	473 (13%)	387 (14%)	64 (12%)	22* (14%)
	All visits (n= 1,731) n %	Private (n= 1,058) n %	Medicaid (n= 558) n %	Uninsured (n= 115) n %
Tobacco smoking cessation	314 (18%)	204 (19%)	88 (16%)	22* (19%)

Table 4 displays the weighted proportion of visits for each type of preventive service provided across the subpopulations. Specifically, low rates (<5%) were discovered for four screenings: depression, HIV infection, unhealthy alcohol use, and intimate partner violence.

**Table 4:** Likelihood of received services (weighted) The n values for each service correspond to those mentioned in Table 3. The National Ambulatory Medical Care Survey (NAMCS) states for all estimates, other than estimates of proportion, at least 30 observations should be present. CI: confidence interval

Service	% All Visits [95% CI]	% Private [95% CI]	% Medicaid [95% CI]	% Uninsured [95% CI]
High blood pressure	94.3% [92.8, 95.5]	95.0% [93.8, 96.0]	92.3% [87.5, 95.3]	92.2% [85.5, 96.0]
Depression	4.5% [3.4, 5.8]	5.0% [3.8, 6.6]	3.1% [1.8, 5.3]	0.8%* [0.3, 2.3]
HIV infection	3.8% [2.5, 5.6]	3.5% [2.1, 5.9]	4.9% [2.8, 8.4]	0.2%* [0.05, 0.9]
Unhealthy alcohol use	2.6% [1.6, 4.2]	3.1% [1.8, 5.1]	1.4%* [0.6, 2.8]	0.8%* [0.3, 2.5]
Cervical cancer	23.6% [21.6, 25.8]	27.0% [24.7, 29.3]	13.5% [9.8, 18.2]	12.2% [8.2, 17.7]
Intimate partner violence	2.2% [1.0, 4.9]	2.6% [1.0, 6.4]	1.4%* [0.6, 3.6]	0.9%* [0.2, 3.7]
Weight loss to prevent obesity-related morbidity and mortality	14.1% [11.9, 16.7]	15.0% [12.5, 17.9]	11.2% [7.1, 17.3]	8.3%* [4.2, 15.7]
Tobacco smoking cessation	16.6% [13.6, 20.2]	19.3% [15.4, 24.1]	11.6% [8.1, 16.4]	13.2%* [6.9, 23.8]

Table 5 shows the odds ratios of services received by patient insurance type. Due to low rates of delivery for some of the preventive services, stable estimations could not be made for all services examined. The study found the odds of Medicaid office visits receiving cervical cancer screenings and tobacco smoking cessation screenings to be 47% and 31% lower, respectively, than those of visits by private insurance patients. Additionally, the study found the odds of receiving blood pressure screenings and cervical cancer screenings to be 43% and 62% lower, respectively, in uninsured office visits compared to private insurance. Unadjusted odds ratios are depicted in Table 5, and odds ratios adjusted for sex, gender, race, and age are depicted in Table 6.

**Table 5:** Odds of received services (no controls) Productivity visits are the reference group. Values represent the odds of receiving each service in relation to productivity-based physicians. An odds ratio comparing salary visits to mixed visits, with salary as the reference. †p < 0.15 ††p < 0.10 †††p < 0.05 The National Ambulatory Medical Care Survey (NAMCS) states for all estimates, other than estimates of proportion, at least 30 observations should be present. CI: confidence interval

Service	Medicaid [95% CI]	Private [Reference= 1.0]	Uninsured [95% CI]
High blood pressure	0.75 [0.50, 1.11]	–	0.56^†††^ [0.36, 0.87]
Depression	0.62^†††^ [0.39, 0.99]	–	*0.34^†††^ [0.18, 0.66]
HIV infection	1.51 [0.82, 2.75]	–	*0.34^†††^ [0.14, 0.83]
Unhealthy alcohol use	*0.43^††^ [0.18, 1.02]	–	*0.26^††^ [.08 , 0.90]
Cervical cancer	0.42^†††^ [0.29, 0.61]	–	0.38^†††^ [0.24, 0.58]
Intimate partner violence	*0.54 [0.16, 1.76]	–	*0.34 [0.06, 1.88]
Weight loss to prevent obesity-related morbidity and mortality	0.72 [0.42, 1.23]	–	*0.51^††^ [0.24, 1.11]
Tobacco smoking cessation	0.55^†††^ [0.34, 0.88]	–	*0.63 [0.33, 1.23]

**Table 6:** Odds of received services (controlled) Controls: sex, gender, race, and age. Productivity visits are the reference group. Values represent the odds of receiving each service in relation to productivity-based physicians. An odds ratio comparing salary visits to mixed visits, with salary as the reference. †p < 0.15 ††p < 0.10 †††p < 0.05 The National Ambulatory Medical Care Survey (NAMCS) states for all estimates, other than estimates of proportion, at least 30 observations should be present. CI: confidence interval

Service	Medicaid [95% CI]	Private [Reference= 1.0]	Uninsured [95% CI]
High blood pressure	0.74 [0.47, 1.15]	–	0.57^†††^ [0.37 0.88]
Depression	0.80 [0.49, 1.30]	–	*0.36^†††^ [0.19, 0.69]
HIV infection	0.91 [0.53, 1.55]	–	*0.27^†††^ [0.11, 0.68]
Unhealthy alcohol use	*0.60 [0.20, 1.85]	–	*0.27^†††^ [.08, 0.94]
Cervical cancer	0.53^†††^ [0.38, 0.73]	–	0.38^†††^ [0.24, 0.60]
Intimate partner violence	*0.61 [0.19, 1.97]	–	*0.35 [0.06, 1.95]
Weight loss to prevent obesity-related morbidity and mortality	0.75 [0.42, 1.32]	–	*0.56^†^ [0.27, 1.18]
Tobacco smoking cessation	0.69^†^ [0.44, 1.08]	–	*0.66 [0.36, 1.24]

Finally, this study showed office visit duration varied according to type of insurance (Table 2). Overall, the average visit duration across all visits was 21.4 minutes. Physicians spent the most time during office visits with private insurance patients, averaging 22.0 minutes, followed by uninsured patients, who averaged 20.1 minutes. Physicians spent the least amount of time during office visits with Medicaid patients, averaging 19.4 minutes. The weighted difference in office visits was -2.68 minutes for Medicaid and -1.97 minutes for the uninsured compared to visits by patients with private insurance.

## Discussion

The purpose of this study was to analyze the association between the type of insurance and the delivery of preventive care services during preventive care office visits. The analysis showed a variance in the rates of preventive service delivery by the type of insurance seen in-office visits. This study found the odds of receiving preventive care to be higher with private insurance when compared to Medicaid and uninsured office visits. Private insurance office visits showed higher odds of receiving cervical cancer screenings and tobacco smoking cessation counseling compared to Medicaid office visits. Additionally, private insurance office visits showed higher odds of receiving high blood pressure screenings and cervical cancer screenings compared to uninsured office visits. Nearly every preventive service observed in this study showed significantly lower odds of provision in uninsured office visits compared to private insurance; however, because the uninsured sample size lacked sufficient statistical power, stable estimates could not be inferred. Future studies with greater statistical power are needed to determine the odds of receiving preventive services when comparing private insurance to uninsured office visits.

This study showed differences in the delivery of preventive services during office visits based on the type of insurance. The findings from this study support our hypothesis that the delivery of preventive care by physicians has a financial component. This study showed that private insurance office visits had the highest odds of providing preventive care. This insurance type also provides the highest reimbursement rates. This hypothesis is also supported by a previous study that showed the delivery of preventive care during office visits varied by financial compensation model [15]. That study showed physicians compensated by productivity-based measures were less likely to provide preventive services compared to physicians compensated by salary or mixed (salary plus productivity-based) models.

Another significant finding from this study was the difference in average visit duration among the different insurance types. Office visits with private insurance averaged longer durations compared to those with Medicaid and the uninsured. This finding aligns with other results in this study, specifically the lower rates of preventive care provided during both Medicaid and uninsured office visits. This supported our hypothesis that physicians compensated by Medicaid reimbursement rates or no reimbursement (uninsured) spend less time during office visits and provide fewer preventive care services. More recent studies are necessary to identify the impact of the ACA on preventive care delivery. While the ACA removed patients’ financial barriers to receiving preventive services by waiving copayments and coinsurance, differences in reimbursement rates by insurance may still result in variation in the provision of preventive services.

This study was limited by the small number of observations with specific types of preventive services. While the sample size was large enough to conduct analyses, some relatively large effect sizes did not reach statistical significance due to limited statistical power. While NAMCS data are designed to make the study nationally representative, several preventive services were provided so infrequently that stable estimates could not be produced. The data set used (NAMCS) is also at the office visit level, and caution should be taken when extrapolating findings made from this data set because they may not be generalizable to individual patients or physicians. Additionally, while this study focused on 'A' and 'B' recommendations from the USPSTF, there are other preventive screening recommendations suggested by other agencies of high importance that were not part of this analysis.

The preventive services examined in this study all had an ‘A’ or ‘B’ grade and are recommended during office visits. With ‘A’ or ‘B’ graded recommendations, there is a high certainty that the net benefit for providing the service is moderate to high; however, literature has shown providers are not delivering these services, with this study supporting the hypothesis that this might be due to financial considerations [1,14]. Recommendations set forth by public health services, such as the United States Preventive Services Task Force (USPSTF), are meant to be implemented during preventive care office visits. By implementing these recommendations, providers can preserve the health of their patients and reduce the incidence and burden of chronic disease [5,7].

## Conclusions

As the future of healthcare systems in the United States evolves with the implementation of different health management systems and reimbursement structures, preventive care belongs at the forefront of change. The low rates of preventive care observed in this study were alarming and can hopefully encourage providers to implement preventive care more often in their clinics. Future studies in this field could focus on why physicians are not providing higher rates of preventive care and how reimbursement rates can be used to improve the provision of preventive care. By following USPSTF guidelines, physicians can increase the quality of care they deliver, increase the longevity of their patients, and reduce healthcare costs in the United States.
